# Microbially produced imidazole propionate impairs prostate cancer progression through PDZK1

**DOI:** 10.1186/s10020-025-01073-0

**Published:** 2025-01-16

**Authors:** Shengkai Jin, Yuhua Zhou, Jing Lv, Yichen Lu, Yuwei Zhang, Menglu Li, Ninghan Feng

**Affiliations:** 1 Wuxi School of Medicine, Jiangnan University, Wuxi, 214122 China; 2 Nanjing Medical University, Nanjing, 211166 China; 3 Nantong University Medical School, 9 Qiangyuan Road, Nantong, 226019 China; 4 Department of Urology, Jiangnan University Medical School, Jiangnan University Medical Center (Wuxi No. 2 People’s Hospital), 68 Zhongshan Road, Wuxi, 214002 Jiangsu China

**Keywords:** Imidazole propionate, Histidine, Prostate cancer, Microbiome, Castration-resistant prostate cancer

## Abstract

**Background:**

A close relationship exists between castration-resistant prostate cancer (CRPC) and histidine metabolism by gut microbes. However, the effects of the histidine metabolite imidazole propionate (IMP) on prostate cancer (PCa) and its underlying mechanisms are not well understood.

**Methods:**

We first assessed the effects of IMP on cell proliferation and migration at the cellular level. Subsequently, we investigated the mechanism of action of IMP using transcriptome sequencing, qPCR, and Western blot analysis. Finally, we validated our findings in vivo using a mouse model.

**Results:**

Histidine had no effect on PCa cell proliferation; however, IMP significantly inhibited the proliferation and migration of PC3 and DU145 cells. Mechanistic studies indicate that IMP exerts its effects by upregulating PDZK1 expression, which subsequently inhibits the phosphorylation of the PI3K-AKT pathway.

**Conclusions:**

In conclusion, IMP significantly inhibits the progression of PCa, offering new insights into potential treatments for CRPC.

**Supplementary Information:**

The online version contains supplementary material available at 10.1186/s10020-025-01073-0.

## Background

Prostate cancer (PCa) is a commonly diagnosed cancer in men, which has approximately 1,410,000 new cases and 375,000 deaths worldwide (Pejcic et al. 2023; Sung et al. 2021). The incidence and mortality of PCa are still increasing year by year. Currently, clinical treatment options for PCa include endocrine therapy, chemotherapy, and surgery, especially localized PCa can be cured by radical prostatectomy (Han et al. 2023; Chandrasekar et al. 2015). However, once the tumor has metastasized (i.e., bone), PCa is considered incurable and become the leading cause of death, which greatly reduces the lifespan and quality of life of patients (Cai et al. 2023; Rebello et al. 2021; Siegel et al. 2020). Among them, androgen deprivation therapy (ADT) is the mainstay of treatment for advanced PCa (Moul 2004; Mehta et al. 2021). However, ADT is still associated with poor prognosis in some patients with advanced PCa, particularly in cases of CRPC that are insensitive to ADT (Pernigoni et al. 2021). Therefore, there is a need to find more effective drugs or combination methods.

Histidine (His) metabolism is a complex biochemical process, which could converted His into various derivatives (Petrova et al. 2020; Chioccioli et al. 2020). His metabolic pathways are potentially relevant to a variety of diseases, e.g., metabolic syndrome, inflammatory bowel disease, and cancer (Holecek 2020). For example, Zhang et al. reported that His metabolism affects the level of histidine in the serum of CRPC patients and found that the level of histidine in the serum decreased significantly with the progression of PCa (Zheng et al. 2020). Imidazole propionate (IMP), as the end product of His metabolism, also reveals the development of the disease to a certain extent. Although the effects of IMP on tumors remain unclean, Chen et al. have reported that IMP could inhibit the NF-κB pathway (Matsushita et al. 2021). Additionally, Zhong et al. have found that activation of the NF-κB pathway could enhance PCa progression and contribute to drug resistance (Seidel et al. 2022; Zhong et al. 2022). However, the effects of IMP on CRPC remin unclean and require further investigation.

In our study, we demonstrated the effect of IMP on advanced PCa and confirmed that the anti-proliferative and anti-migratory effects of IMP on CRPC. We first examined the effect of IMP on PCa cells via in vitro experiments. Subsequently, to further ascertain the mechanism of action of IMP on CRPC cells, transcriptomics sequencing and western blot results showed that IMP upregulated PDZK1 expression and inhibited PI3K-AKT activation. Finally, in animal experiments, we found that IMP could significantly inhibits the progression of CRPC. This study provides new evidence of the role of IMP in cancer and offers a new approach to the clinical treatment of advanced PCa.

## Materials and methods

### Ethical statement and tissue collection

This study was approved by the Ethics Committee of Wuxi No. 2 Hospital affiliated with Nanjing Medical University (2022-Y-80). The PCa specimens and their paired normal tissues were obtained from patients at Wuxi No. 2 Hospital and all participants have signed informed consent forms. All patients received no endocrine therapy before surgery, and all patients underwent radical prostatectomy. The tissues were immediately preserved in liquid nitrogen.

### Cell culture

Human prostate cancer cell lines PC3 (CL-0185, Procell), DU145 (CL-0075, Procell), LNCap (CL-0143, Procell) and 22RV1 (CL-0004, Procell) were maintained in F12K medium (PYG0036, Boster) supplemented with 10% foetal bovine serum (FBS) (CTCC-002-071-50, Meisen), pencicillin (100 U/ml) and streptomycin (100 U/ml) (15140122, Gibco). Human normal prostate epithelial cells RWPE-1(CTCC-003-0013, Meisen) were maintained in RPMI 1640 medium (PYG0006, Boster) supplemented with 10% FBS (CTCC-002-071-50, Meisen), pencicillin (100 U/ml) and streptomycin (100 U/ml) (15140122, Gibco). All the cells were culture at 37℃ in a humidified incubator with 5% CO_2_.

### Reagents

Imidazole propionate was purchased from MedChemExpress (HY-W045271). L-Histidine was purchased from MedChemExpress (HY-N0832). Trypsin-EDTA was purchased from Gibco (25200072). Cellsaving was purchased from NCM Biotech (C40100). Cell counting kit-8 (CCK8) was obtained from DOJINDO (CK04). 4% Paraformaldehyde Fix Solution was obtained from Beyotime (P0099). Phosphate buffered solution (PBS) was obtained from Biosharp (BL302A). Methyl Violet was obtained from Aladdin (M112775). BeyoClick™ EdU Cell Proliferation Kit with Alexa Fluor 488 was obtained from Beyotime (C0071S). Antibodies against PI3 Kinase p85 was purchased from Cell Signaling Technology (#4257). Phospho-PI3K p85 alpha (Tyr607) Antibodies was was purchased from Affinity Biosciences (AF3241). AKT Polyclonal antibody and Phospho-AKT (Ser473) Polyclonal antibody was purchased from proteintech (10176-2-AP, 28731-1-AP). mTOR Monoclonal antibody was purchased from proteintech (66888-1-lg), phospho-mTOR (Ser2448) was purchased from Cell Signaling Technology (#2971), Anti-PDZK1 antibody was purchased from UpingBio (YP-53b-01734). Protein Free Rapid Sealing Solution was purchased from Boster (AR0041). Skim milk power was purchased from Biosharp (BS102). NcmBlot Rapid Transfer Buffer (20X) was purchased from NCM biotech (WB4600). Primary Antibody Dilution Buffer was purchased from Beyotime (P0023A). QuickBlock™ Secondary Antibody Dilution Buffer for Western Blot was purchased from Beyotime (P0258). NcmECL Ultra was purchased from NCM biotech (P10200). Bicinchoninic acid (BCA) Protein Assay Kit was purchased from Beyotime (P0012).

### Cell viability assay

The inhibitory effect of imidazole propionate on the proliferation of PC3, DU145 and LNCap and 22RV1 cells was determined by CCK8 assay as we described previously. The cells were seeded in 96-well plates at densities of 5 × 10^3^ cells per well. After 24 h incubation, the three cell lines were treated with different concentrations of IMP for 24–48 h. Subsequently, 10 µL of CCK8 reagent was added to each well, according to the manufacturer’s instructions. After incubation at 37 ℃ for 2 h, optical density was measured at 450 nm.

### Colony formation

PC3 and DU145 cells were seeded at 1000/well into 6-well plates for colony formation. After approximately 1 week of culture, cells were fixed with 4% paraformaldehyde for 30 min and stained with 0.1% crystal violet for 20 min at room temperature in the dark. Visible colonies (diameter > 0.1 mm) were photographed and counted.

### Wound healing assay

The migration capacity of PCa cells was first determined by wound healing assay. Briefly, PC3 and DU145 cells were seeded onto 6-well plates and grown until a confluent monolayer was formed. Use a sterile pipette gun pipette tip to scratch out a wound and then wash once using PBS. Add IMP diluted in medium without FBS put back into the cell incubator to continue incubation for 24 h and images were captured.

### EdU cell proliferation assay

PC3 cells (1 × 10^4^) and DU145 cells (1 × 10^4^) were cultured in 6-well plates for 24 h and then IMP was added to continue the incubation for 48 h. EdU was diluted with cell culture medium at 1:500, and an equal volume of diluted EdU was added to the 6-well plates to make the final concentration of 10 µM. The 6-well plates were gently shaken well and then placed into the cell culture incubator to continue incubating the cells for 2 h. EdU labeling was performed. After the cells were finished, the culture solution was removed and 1 ml of 4% paraformaldehyde was added and fixed for 15 min at room temperature. After removing the fixative, the cells were washed with 1 ml of washing solution per well. The washing solution was removed and each well was incubated with 1 ml of permeabilization solution (PBS containing 0.3% Triton X-100) for 10–15 min at room temperature. After removing the permeabilizing solution and washing again, an appropriate amount of click reaction solution was added to each well and incubated at room temperature away from light for 30 min. After removing the click reaction solution, DAPI was added and incubated, images were acquired.

### Transwell migration assay

This assay was performed using Transwell Boyden chambers containing a polycarbonate membrane (8.0 µM, pore size, Corning, NY, US). A total of 2 × 10^4^ cells in 100 µl of serum-free medium was plated in the upper wells of Boyden chambers, and the bottom wells were filled with 650 µl of culture medium supplemented with 10% FBS as a chemoform. The same concentrations of IMP were added to both the upper and lower compartments. After 48 h incubation, the nonmigrated PC3 and DU145 cells on the upper surfaces were removed, and the migrated cells on the lower surfaces were fixed with 4% paraformaldehyde and stained with crystal violet for 30 min. The migrated cells per chamber were counted in three randomly selected fields, and Images were acquired using a brightfield microscope (100x magnification), and cells were counted by ImageJ (version 1.54). Images were acquired using a brightfield microscope (100x magnification), and cells were counted by ImageJ (version 1.54).

### Total RNA extraction and quantitative real-time PCR (qRT–PCR)

Total cellular RNA was extracted using FastPure^®^ Cell/Tissue Total RNA Isolation Kit (RC112-01, Vazyme). Then performed with HiScript III RT SuperMix (R323-01, Vazyme) for mRNAs. qRT-PCR was then performed using ChamQ Universal SYBR qPCR Master Mix (Q711-02, Vazyme) and an Applied Biosystems QuantStudio 5 system (A28569, Thermo Scientific). Real-time PCR was performed using 1X SYBR Green Master Mix using the primers in Table S1.

### Western blot analysis

Selected PC3, DU145, LNCap and 22RV1 cells in logarithmic growth phase were digested with trypsin and resuspended and counted and spread evenly in 6-well plates at 1 × 10^5^/ml. After incubation with IMP for 24 h/48 h, total proteins were extracted using radioimmunoprecipitation (RIPA) lysis buffer (P0013B, Beyotime) containing protease inhibitor cocktail (P1082, Beyotime). A BCA protein assay kit was used to detect the protein concentration. Equal amounts of protein (50 µg) were separated by 10–12% SDS-PAGE and then transferred to PVDF membranes. The membranes were then immersed in 5% skim milk for 1 h at room temperature. Subsequently, membranes were incubated with primary antibodies at 4℃ overnight, washed with TBST solution and incubated with HRP-tagged secondary antibody for 1 h at room temperature. Finally, the Western blot bands were visualized using enhanced chemiluminescence (ECL) solution.

### In vivo experiments

This study was reviewed and approved by the Animal Protection and Jiangsu Provincial Ethics Committee of Jiangnan University (#JN. No 20230830b0161215 [340]). Healthy 6-week-old BALB/c nude mice were purchased from Nanjing Medical University (Nanjing, China) and randomly divided into four groups of five mice each. Approximately 5 × 10^6^ PC3 cells washed with PBS were injected at one time point into the blood-rich area in the mid-posterior region of the axilla of the left forelimb of each nude mouse. Tumor nodules and progression were monitored every 7 days after injection. The tumor volume measurement formula is as follows: volume = (length × width^2^)/2. Five weeks after injection, all nude mice were executed under general anesthesia (induced by intraperitoneal injection of sodium pentobarbital (150 mg/kg)) and the tumors were removed for subsequent analysis. For drug treatment, 40 mg IMP in 1 mL containing 1% DMSO was dissolved and injected intraperitoneally into nude mice every two days.

### Statistical analysis

Prism statistical software (GraphPad 9.0, San Diego, CA, US) was employed for statistical analysis. The values are expressed as the mean ± SEM of three independent experiments. Student’s t-test was used for comparing the differences of two groups. Data between multiple groups were compared by one-way ANOVA. *P* < 0.05 was considered significant.

## Results

### IMP inhibits the proliferation of PC3 and DU145

**Fig. 1:**
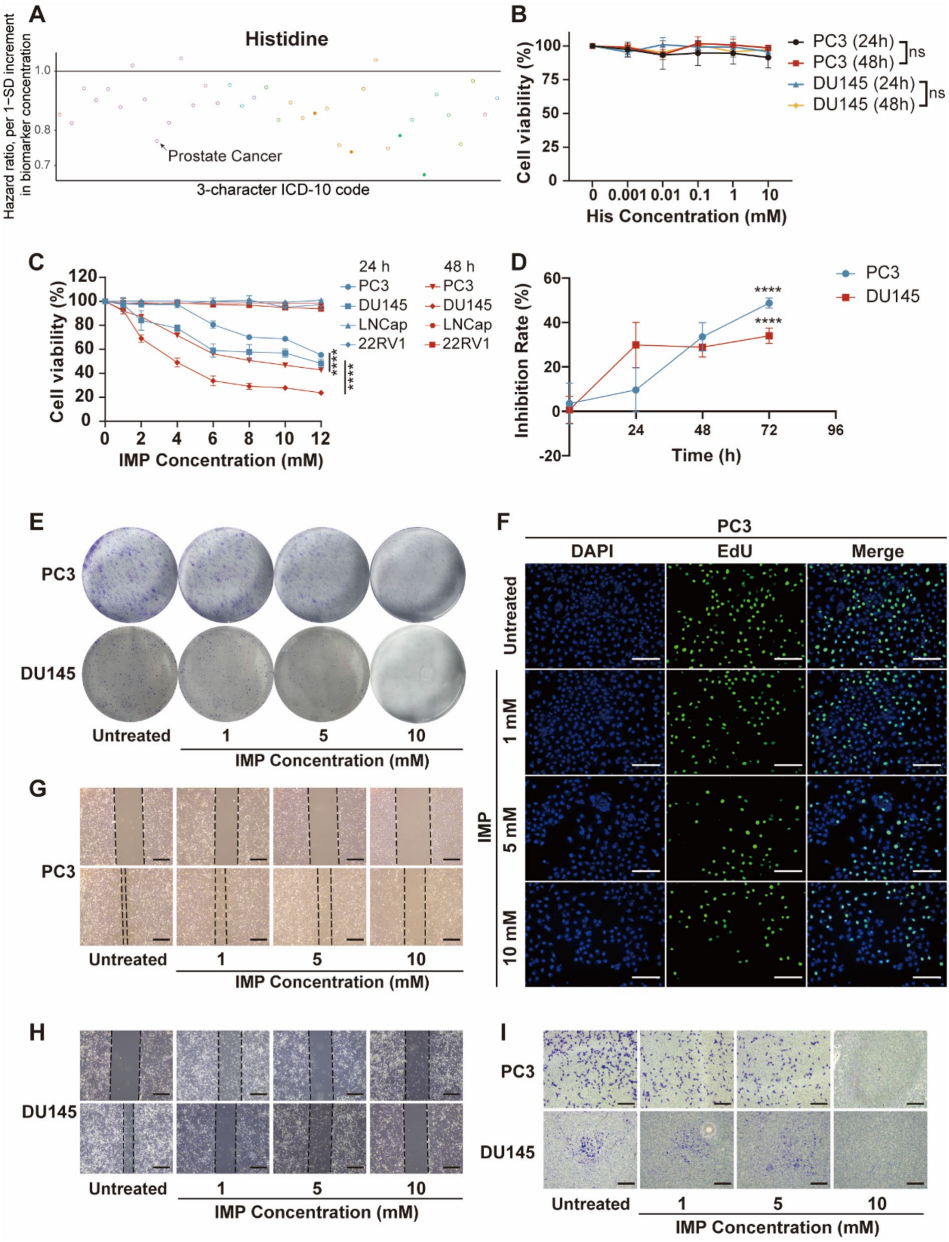
IMP could inhibit the proliferative and migratory activities of PCa. **A** Analysis of histidine and risk of death from PCa. **B** Cell viability assay of PC3 and DU145 cells following HIS stimulation. **C** Cell viability assay of PC3, DU145, LNCap and 22RV1 cells after IMP stimulation. **D** Inhibition rate of IMP (10 mM) on PC3 and DU145 cells at different times. **E** Cell colony formation of PC3 and DU145 cells with IMP stimulation. **F** EdU staining plots of PC3 cells after IMP stimulation. Scale bar, 200 μm. **G** Wound healing diagram after IMP stimulation of PC3 cells. Scale bar, 500 μm. **H** Wound healing diagram after IMP stimulation of DU145 cells. Scale bar, 500 μm. **I** Transwell migration plots after IMP stimulation of PC3 and DU145 cells. Scale bar, 200 μm. *P < 0.05, **P < 0.01, ***P < 0.001, ****P < 0.0001; ns, not significant

To demonstrate the relationship between His metabolic and PCa, we conducted an analysis of the correlation between His levels and PCa using a comprehensive biomarker-disease association atlas (available at nightingalehealth.com/atlas). The results showed that His concentration was negatively correlated with PCa mortality [Hazard ratio (HR) = 0.768, *P* = 2.48E−05] (Fig. 1A and Table S2). Additionally, we treated two commonly used CRPC cell lines (PC3 and DU145) with His for 24 h and 48 h, respectively, as a way to determine the exact relationship between His and CRPC. The results of CCK8 assay showed that the cell viability of both cell lines remained above 75% with the increment of His concentration (Fig. 1B). We therefore speculate that the negative correlation between His and PCa may be related to its metabolites.

Subsequently, we investigated whether IMP was able to influence the proliferation of PCa cells using a cell viability assay to determine whether IMP was able to influence the proliferation of PCa cells. The results showed that the viability of PC3 and DU145 cells decreased significantly with increasing IMP concentration (Fig. 1C). Nevertheless, the viability of LNCap and 22RV1 cells remained above 80% throughout the experimental period. Subsequently, we investigated whether the cellular effects of IMP on CRPC were time-dependent. The results demonstrated that the inhibitory effects of IMP on PC3 and DU145 reached 48.76% and 34.06%, respectively, when IMP treatment was extended to 72 h (Fig. 1D).

Furthermore, we carried out a colony formation assay to determine the effect of IMP on the colony forming ability of CRPC cells. After 48 h of treatment, the number and size of colonies were significantly reduced with increasing IMP concentration in the IMP-treated group compared to the untreated group, suggesting that IMP inhibits the tumorigenic capacity of PC3 and DU145 cells in vitro (Fig. 1E and Figure S1B). This finding prompted us to assess the impact of IMP on the proliferative activity of PC3 and DU145 in greater depth. Due to the decreased cell proliferative activity, which results in the difficulty of incorporating EdU into DNA, we investigated the impact of IMP on the proliferative ability of PC3 and DU145 cells through the use of EdU (Radwan et al. 2022). The results demonstrated that the PC3 and DU145 cell lines exhibited a significantly reduced number of EdU-stained cells following treatment with high concentrations of IMP, in comparison to the control group (Fig. 1F, Figure S1C and S1D). Overall, these findings emphasize the effective inhibitory effect of IMP on the proliferation of PC3 and DU145 cells. In addition, we observed that the IMP was administered at a higher concentration, therefore we conducted a safety assessment of IMP. The results showed that the cell viability of RWPE-1 cells remained above 70% even at IMP concentrations up to 12 mM, indicating that IMP is less toxic to normal cells (Figure S1A). We also used Annexin-V-FITC/PI double staining and flow cytometry analysis to verify whether IMP induced apoptosis in PC3 and DU145 cells. Apoptosis in PC3 and DU145 cells did not change significantly after IMP treatment (Figure S1F). Taken together, these findings suggest that IMP has the ability to inhibit the proliferation of CRPC cells.

### IMP inhibits the migration of PC3 and DU145

To investigate the effects of IMP on CRPC migration, we use IMP treatment of PC3 and DU145 for cell migration experiments. In wound healing experiments, the healing area of cellular wounds decreased with increasing treatment concentrations. Specifically, at 10 mM, the healing rates of PC3 and DU145 cells were reduced to 44.19% and 6.13%, respectively (Fig. 1G, H and Figure S2A). Due to the inability of the scratch assay to effectively distinguish between cell migration and proliferation, we employed the Transwell migration assay for our studies. As shown in Fig. 1I and Figure S2B the number of cells migrating through the membrane decreased to varying extents following IMP treatment at concentrations of 1, 5, and 10 mM. This finding is consistent with the wound healing assay results, which demonstrated a reduction in the migratory ability of PC3 and DU145 cells after IMP treatment. Collectively, these results suggest that IMP inhibits migration of CRPC cells in a concentration-dependent manner.

**Fig. 2:**
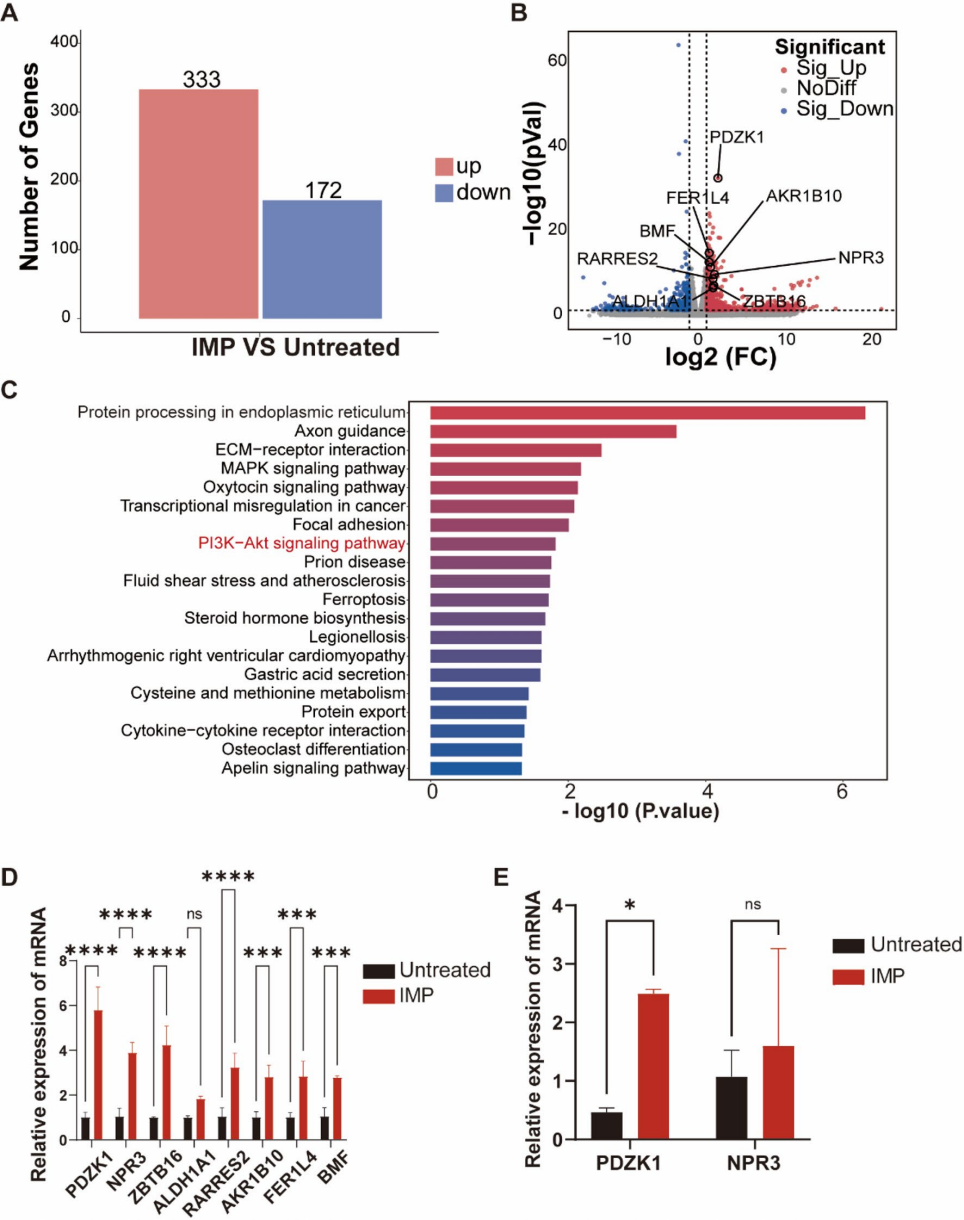
IMP could alter gene expression in PCa and result in the upregulation of PDZK1 expression. **A** Number of differentially expressed upregulated (log2FC ≥ 1 & q < 0.05) and downregulated (log2FC ≤ −1 & q < 0.05) genes in each comparison group. **B** Location of the PDZK1 gene in the volcano map. **C** The top 20 KEGG pathways with the smallest P-value (or Q-value) were utilized for mapping, with the vertical coordinate being the pathway name and the horizontal coordinate being the − log10 value of the P-value or Q-value of the KEGG pathway enrichment analysis for that KEGG pathway. **D** Histogram depicting mRNA expression levels in PC3 cells. **E** PDZK1 and NPR3 mRNA levels in PC3 cells. *P < 0.05, **P < 0.01, ***P < 0.001, ****P < 0.0001; ns, not significant

### IMP exerts inhibitory effects on PCa cell phenotype via PDZK1

**Fig. 3:**
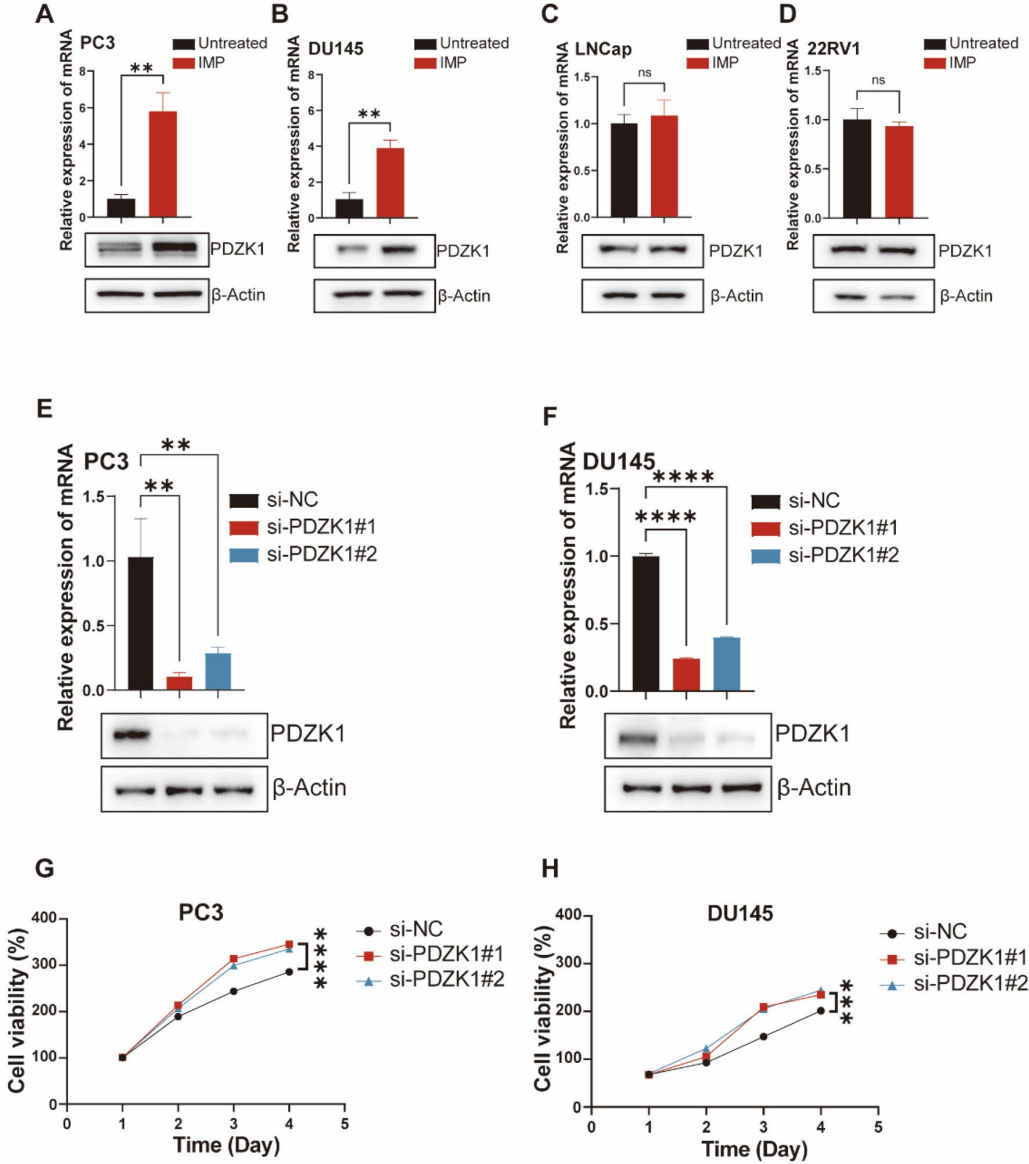
Knockdown of PDZK1 affects the proliferative ability of cells. **A** PDZK1 mRNA expression levels and protein expression levels in PC3. **B** PDZK1 mRNA expression levels and protein expression levels in DU145. **C** PDZK1 mRNA expression levels and protein expression levels in LNCap. **D** PDZK1 mRNA expression levels and protein expression levels in 22RV1. **E** Knockdown efficiency of PDZK1 in PC3. **F** Knockdown efficiency of PDZK1 in DU145. **G** Cell viability assay after PC3 knockdown of PDZK1. **H** Cell viability assay after DU145 knockdown of PDZK1. *P < 0.05, **P < 0.01, ***P < 0.001, ****P < 0.0001; ns: not significant

We investigated the potential mechanisms by which IMP exerts its antiproliferative and antimigratory effects in CRPC cells. Transcriptome analysis revealed that IMP treatment significantly up-regulated 333 genes as well as significantly down-regulated 172 genes (Fig. 2A). In order to find the key genes, we performed an initial screen among the more significantly upregulated genes (|log2| (FC) > 1.5, trans_type is protein_codin and DO is associated with cancer). We screened eight genes including PDZK1 and explored them in the volcano map (Fig. 2B). Additionally, Kyoto Encyclopedia of Genes and Genomes (KEGG) enrichment analysis revealed an enrichment of the PI3K-AKT signaling pathway (Fig. 2C). To identify genes with stable differences among the screened candidates, we further assessed the mRNA levels and ultimately selected the PDZK1 gene for detailed analysis (Fig. 2D, E). Increasing evidence suggests that PDZK1 plays a role in inhibiting tumor progression and enhancing sensitivity to chemotherapeutic agents, highlighting its potential as a promising therapeutic target (Ma et al. 2024; Qi et al. 2020; Wang et al. 2024). However, the impact of PDZK1 in PCa represents an intriguing emerging question. We first investigated PDZK1 levels in tumour tissue and normal tissue of PCa patients to study the potential role of PDZK1 in different tissues. tumour tissue exhibited significantly lower levels of PDZK1 protein compared with normal tissue (Figure S1E). Additionally, we evaluated the effect of IMP treatment on PDZK1 protein expression. Specifically, IMP treatment increased the expression level of PDZK1, suggesting that IMP may exert antiproliferative and antimigratory effects by promoting PDZK1 expression (Fig. 3A, B). However, no significant PDZK1 upregulation was observed in LNCap and 22RV1, which may explain the not effective of IMP on androgen-dependent prostate cancer cells (Fig. 3C, D).

To assess the critical role of PDZK1 in PCa cell proliferation and migration, we employed small interfering RNA (siRNA) to knock down PDZK1 expression. Before investigating the role of PDZK1 in cells, we first assessed the efficiency of the siRNA-mediated knockdown. The results demonstrated that the knockdown of PDZK1 led to a significant reduction in both mRNA and protein levels in the cells (Fig. 3E, F). This suggests that siRNA is effective in knocking down PDZK1. Subsequently, we observed an increase in cell viability after knockdown of PDZK1 expression in the knockdown of PDZK1 cell viability assay (Fig. 3G, H). This suggests that PDZK1 may have the potential to inhibit cell proliferation.

**Fig. 4:**
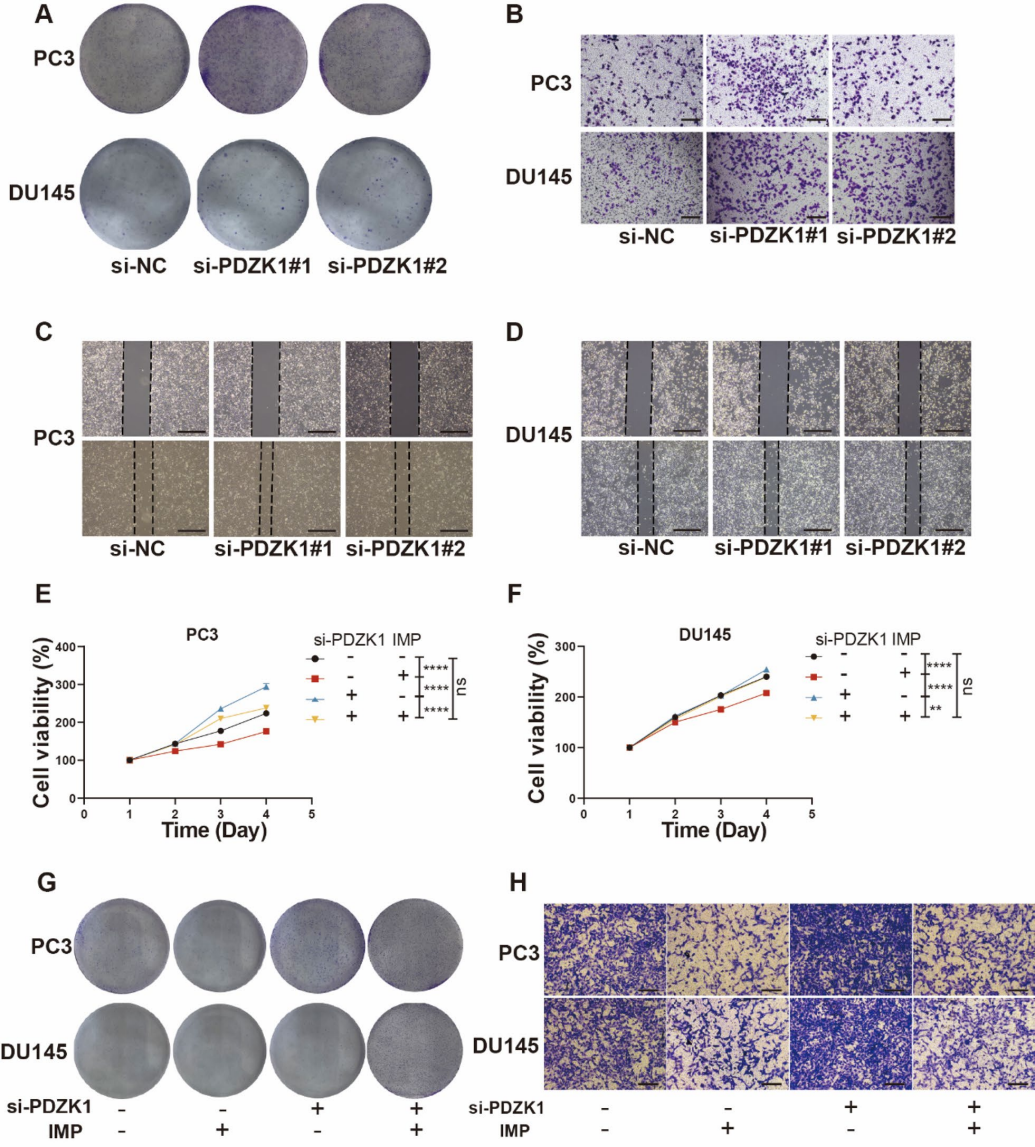
Knockdown of PDZK1 promotes PCa proliferation and migration. **A** Cell colony formation after PDZK1 knockdown in PC3 and DU145 cells. **B** Transwell migration assay after knockdown of PDZK1 in PC3 and DU145 cells. Scale bar, 200 μm. **C** Wound healing assay after knockdown of PDZK1 in PC3 cells. Scale bar, 500 μm. **D** Wound healing assay after knockdown of PDZK1 in DU145 cells. Scale bar, 500 μm. **E** Cell viability assay combining PDZK1 knockdown and IMP treatment using PC3. **F** Cell viability assay combining PDZK1 knockdown and IMP treatment using DU145. **G** Cell clones formed by PC3 and DU145 in a combined PDZK1 knockdown and IMP treatment experiment. **H** Transwell migration experiments of PC3 and DU145 in a combined PDZK1 knockdown and IMP treatment experiment. Scale bar, 200 μm. *P < 0.05, **P < 0.01, ***P < 0.001, ****P < 0.0001; ns, not significant

Additionally, cell cloning experiments demonstrated the impact of PDZK1 knockdown on colony formation, suggesting that PDZK1 plays a critical role in the proliferation of PC3 and DU145 cells (Fig. 4A and Figure S2C). To determine whether PDZK1 affects the migration of PC3 and DU145 cells, we conducted Transwell migration and wound healing assays following PDZK1 knockdown. PDZK1 knockdown resulted in increased cell migration (Fig. 4B-D and Figure S2D, E). These findings suggest that the expression level of PDZK1 plays a critical inhibitory role in the proliferation and migration of CRPC cells.

**Fig. 5:**
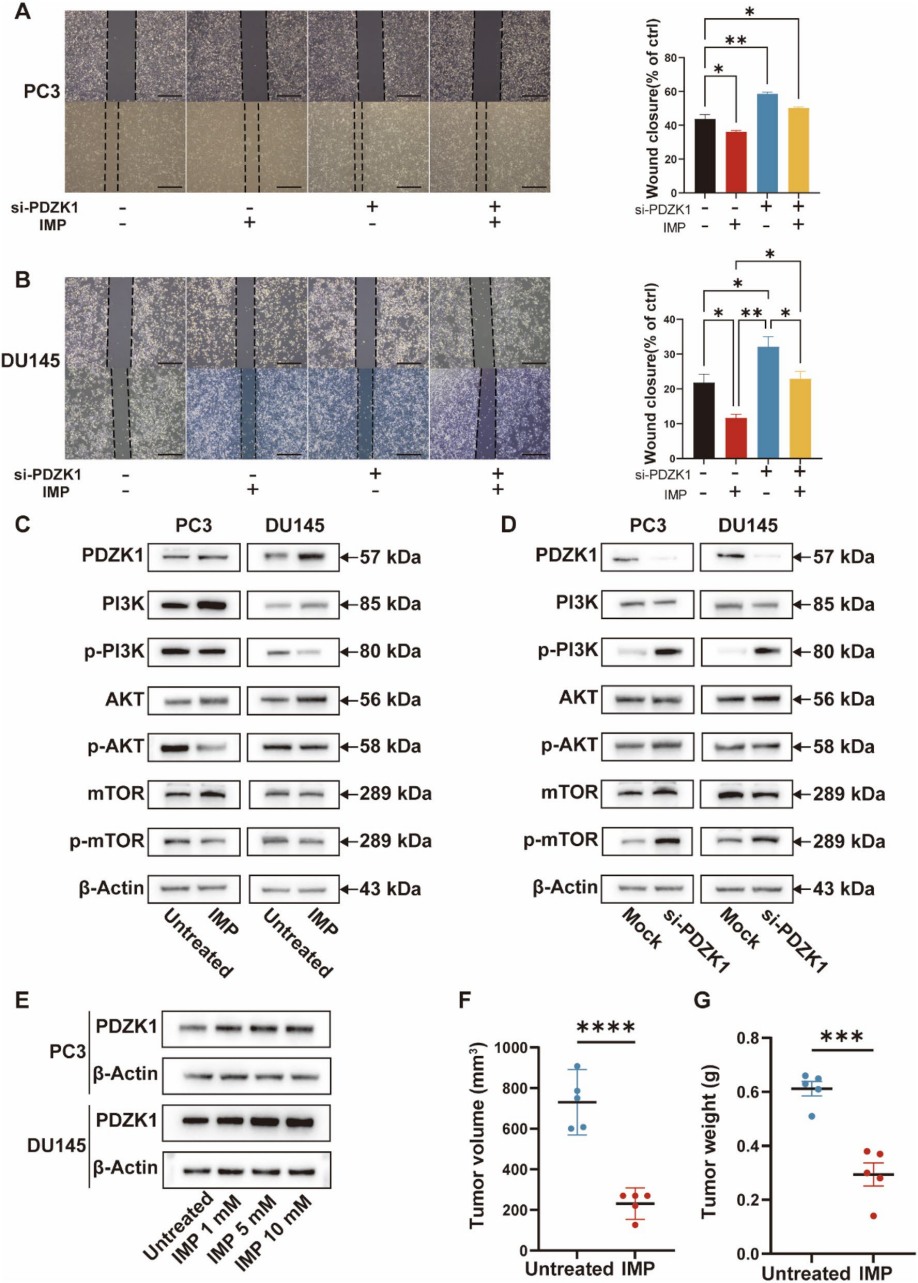
IMP influences PDZK1 protein and PI3K-AKT pathway protein levels in cells. **A** Wound healing assay results (left) and wound healing rate (right) of PC3 cells in a combined PDZK1 knockdown and IMP treatment experiment. Scale bar, 500 μm. **B** Wound healing assay results (left) and wound healing rate (right) of DU145 cells in a combined PDZK1 knockdown and IMP treatment experiment. Scale bar, 500 μm. **C** Representative western blot analysis of PC3 and DU145 cells with or without treatment with IMP (10 mM). **D** Representative western blot analysis of PC3 and DU145 cells with or without si-PDZK1 treatment. **E** PDZK1 protein expression levels in PC3 and DU145 cells treated with different concentrations of IMP. **F** Volumetric and gravimetric analysis of excised tumors (*n* = 5 per group). *P < 0.05, **P < 0.01, ***P < 0.001, ****P < 0.0001; ns: not significant

We defined the potential role of this gene in the process of IMP action through a combination of knockdown and drug treatment experiments. The results showed that si-PDZK + IMP treatment reduced the cell viability growth of si-PDZK-treated cells (Fig. 4E, F). It suggests that IMP may mediate the antiproliferative effect through PDZK1. In the colony formation assay of cells, we observed similar results to those of CCK8. Specifically, si-PDZK1 + IMP treatment increased the number of IMP-treated cell colonies (Fig. 4G and Figure S2F). In the Transwell migration, the si-PDZK1 + IMP group exhibited significantly reduced migration compared to the si-PDZK1 group (Fig. 4H and Figure S2G). In the wound healing rescue experiment, we observed similar results to those in the migration experiment (Fig. 5A, B). Taken together, these data suggest that IMP inhibits the proliferation and migration of PC3 and DU145 cells by upregulating PDZK1 expression.

### IMP inhibits activation of the PI3K-AKT signalling pathway by increasing PDZK1 expression

Transcriptomic analysis revealed that IMP treatment significantly regulated the phosphatidylinositol 3-kinase (PI3K)-protein kinase B (AKT) signalling pathway. Therefore, we examined the effect of IMP on the phosphorylation of this pathway. Specifically, IMP treatment reduced the phosphorylation level of the PI3K-AKT pathway in CRPC cells, suggesting that IMP exerts antiproliferative and antimigratory effects on CRPC by inhibiting the activation of the PI3K-AKT signalling pathway (Fig. 5C and Figure S3A). In addition, we evaluated the impact of PDZK1 knockdown on the phosphorylation of the PI3K-AKT pathway. In contrast to the results observed with IMP treatment, knockdown of PDZK1 led to hyperphosphorylation of the PI3K-AKT pathway (Fig. 5D and Figure S3B). In addition, we observed a progressive increase in PDZK1 protein levels following treatment of PC3 and DU145 cells with increasing concentrations of IMP (Fig. 5E and Figure S2H). This suggests that various concentrations of IMP contribute to the increased expression of PDZK1 and help maintain stable regulation of cell proliferation and migration throughout the experiment. It is well established that phosphorylation of the PI3K-AKT signaling pathway promotes the progression and poor prognosis of PCa. Taken together, these results demonstrate that IMP inhibits PI3K-AKT pathway activation by upregulating PDZK1 expression, which in turn suppresses the proliferation and migration of PC3 and DU145 cells.

### Inhibition of subcutaneous graft tumor growth in nude mice by IMP

**Fig. 6:**
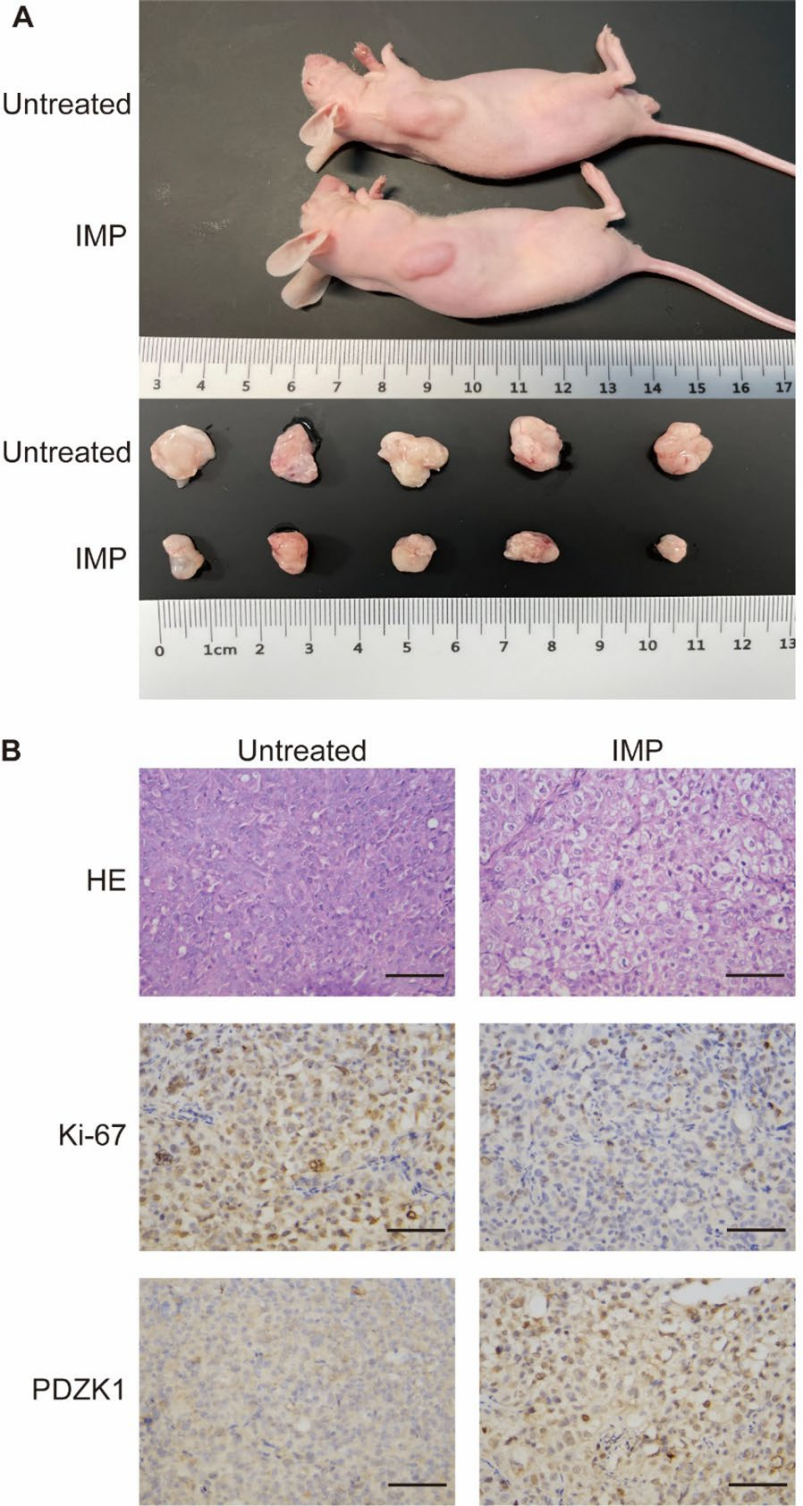
IMP inhibits tumor growth in vivo. **A **In vivo tumor photography and post-mortem tumor imaging after euthanasia of mice. **B** Pathological and immunohistochemical analysis of tumor tissues. Scale bar, 200 μm

To evaluate the in vivo anti-tumor efficacy and safety of IMP, we injected PC3 cells subcutaneously into the right axilla of mice. IMP was administered intraperitoneally every two days starting on the third day post-injection until the tumors became visibly apparent (Figure S3C). We observed that tumor volume and weight in the IMP-treated group were significantly reduced compared to the Control group (Figs. 5F and G and 6A). Additionally, we observed that IMP treatment preserved tumor tissue structure, reduced Ki-67 expression, and increased PDZK1 expression in the tumor tissue (Fig. 6B). Taken together, these findings suggest that IMP possesses significant antiproliferative and antimigratory potential and demonstrates safety in vivo.

## Discussion

**Fig. 7:**
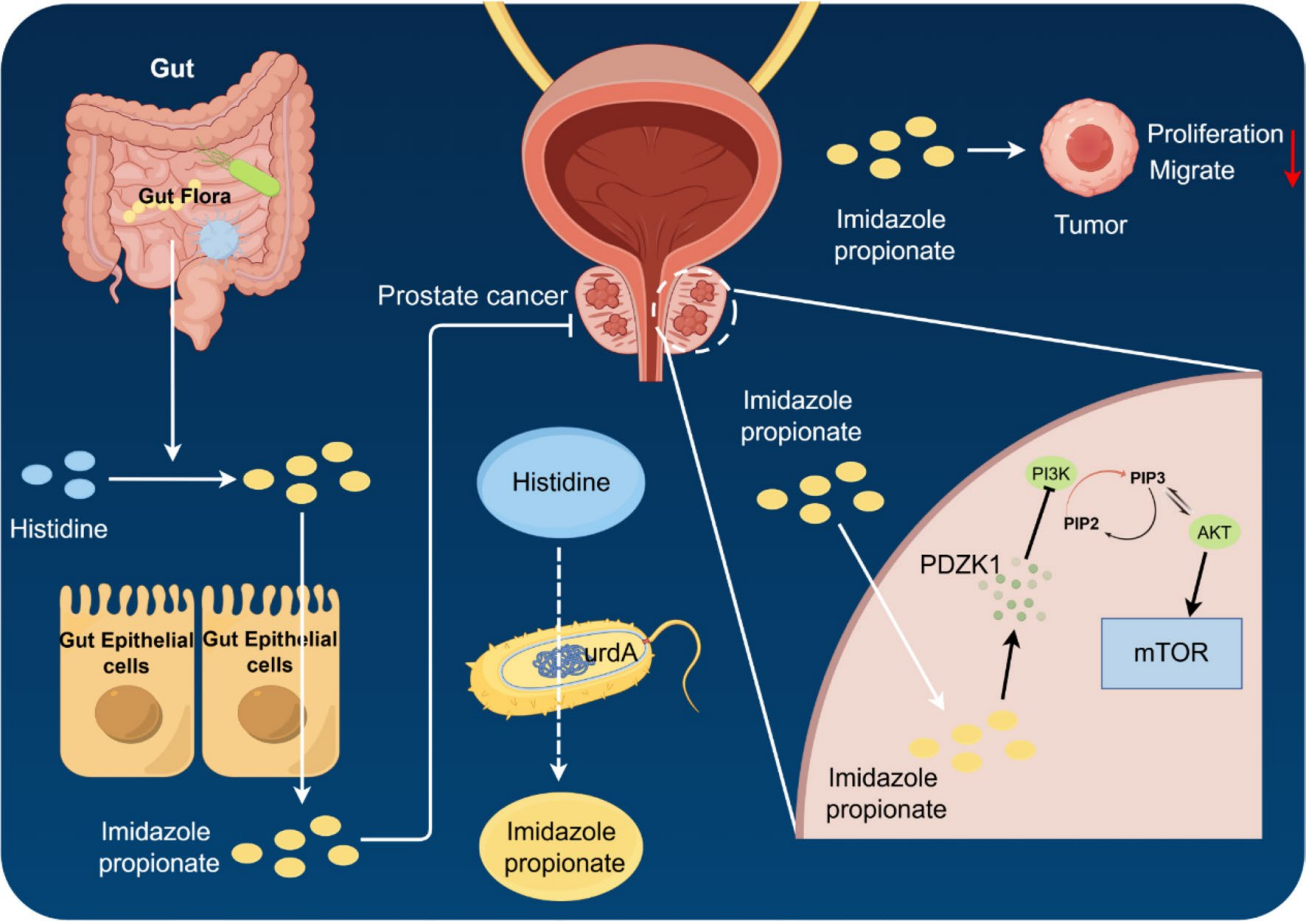
Schematic representation of the mechanism by which IMP inhibits activation of the PI3K-AKT pathway via PDZK1

PCa is one of the most common malignant tumors worldwide and poses a serious risk to human health and the maintenance of public health (Sandhu et al. 2021). Current treatment options for PCa primarily include surgery, chemotherapy, radiotherapy, and immunotherapy; however, the mortality rate among PCa patients continues to rise annually (Bergengren et al. 2023). ADT is considered the primary treatment for PCa, but it is primarily effective only in the early stages of the disease (Dai et al. 2017). The majority of PCa patients treated with ADT experience recurrence and progression to CRPC, which significantly impacts their overall health and quality of life (Pernigoni et al. 2021). Therefore, new and effective treatments for CRPC must be explored.

Dysregulation of the MAPK pathway has been associated with the development of a variety of tumours, such as melanoma, thyroid cancer and ovarian cancer (Ullah et al. 2022). Many studies have now demonstrated that it mainly affects tumour proliferation, survival and metastasis (Guo et al. 2020). However, the PI3K-AKT pathway has an important role in CRPC. Deletion of PTEN in CRPC leads to activation of the PI3K-AKT pathway. Specifically, PI3K converts PIP2 to PIP3, leading to downstream activation of the RAC-α serine/threonine protein kinase (AKT) and rapamycin target protein (mTOR) signalling cascades (Jamaspishvili et al. 2018). Previous studies have reported on the effects of IMP on mTOR. For example, Kim et al. found that IMP significantly inhibits mTORC2, and that activation of mTORC2 promotes the progression of various tumors (Kim et al. 2024; Zhang et al. 2022). Our results suggest that IMP inhibits CRPC progression through exogenous supplementation. Specifically, IMP inhibited the activation of the PI3K-AKT signalling pathway by increasing the level of PDZK1 which in turn inhibited CRPC proliferation and migration (Fig. 7). Our study demonstrated that IMP has a significant inhibitory effect on CRPC cells. Future research should focus on optimizing the efficacy of IMP against CRPC, enhancing its in vivo utilization, and ultimately reducing the mortality rate of CRPC patients.

However, there are certain limitations in this study. First, we demonstrated the inhibitory effect of IMP on CRPC in this study, but the IMP-producing gut microorganisms were not investigated. Previous studies reported that *Brevibacillus laterosporus*, *Adlercreutzia equolifaciens* and *Shewanella oneidensis* could produce IMP by consuming His (Koh et al. 2018). Second, while we explored the correlation between His and PCa, we did not investigate the correlation between IMP and CRPC in clinical samples. To enhance the generalizability of our findings, future studies should strive to include a larger and more diverse cohort. Finally, we need further evaluations to investigate the mechanism of action of IMP on PDZK1.

## Conclusions

Our findings contribute to the understanding of the relationship between His metabolic and PCa, and provide a rational mechanistic basis for exploring therapeutic strategies. Specifically, our findings support the investigation of the clinical antitumor role of gut microbiota metabolites, such as IMP, in tumors characterized by PI3K-AKT pathway activation.

## Supplementary Information

Supplementary Material 1: Figure S1 The effect of IMP on different prostate cells. **A** Safety assay of IMP on normal prostate cancer cells RWPE-1 cells. **B** Histogram depicting cell colony counts following stimulation with IMP for PC3 and DU145 cells. **C** EdU staining plots of DU145 cells after IMP stimulation. Scale bar, 200 μm. **D** Histogram showing EdU detection results for PC3 and DU145 cells. **E** Western blot of PDZK1 in tumour and normal tissues. **F** Apoptosis assay of PC3 and DU145 after 48h of treatment with the corresponding concentrations of IMP. *P < 0.05, **P < 0.01, ***P < 0.001, ****P < 0.0001; ns, not significant.

Supplementary Material 2: Figure S2 Statistical analysis charts. **A** Histogram of wound healing area for PC3 and DU145. **B** Histogram of the number of Transwell migrations. **C** Histogram of cell colony number after knockdown of PDZK1 in PC3 (left) and DU145 (right). **D** Histogram of Transwell mobility after knockdown of PDZK1 in PC3 (left) and DU145 (right). **E** Histogram of wound healing rate after knockdown of PDZK1 in PC3 (left) and DU145 (right). **F** Rate of cell colony formation in rescue experiments with PC3 (left) and DU145 (right). **G** Histogram of Transwell migration of PC3 (left) and DU145 (right) in rescue experiments. **H** PDZK1 protein expression levels grayscale analysis in PC3 (left) and DU145 (right) cells treated with different concentrations of IMP. *P < 0.05, **P < 0.01, ***P < 0.001, ****P < 0.0001; ns, not significant.

Supplementary Material 3: Figure S3 Analysis of grayscale values and protocols for animal experimentation. **A** Quantitative analysis of protein phosphorylation of PC3 (left) and DU145 (right) with or without treatment with IMP (10 mM). **B** Quantitative analysis of protein phosphorylation in PC3 (left) and DU145 (right) cells with or without si-PDZK1 treatment. **C** Schematic of the mouse experiment: On Day 0, PC3 cells were injected subcutaneously. From Day 3, intraperitoneal injections of either IMP or PBS were administered every two days until the tumors became visible (n = 5 per group). *P < 0.05, **P < 0.01, ***P < 0.001, ****P < 0.0001; ns, not significant.

Supplementary Material 4: Table S1. Primer Sequence List.

Supplementary Material 5: Table S2. Histidine and Disease Association Mapping.

## Data Availability

No datasets were generated or analysed during the current study.

## Abbreviations

CRPC: Castration-resistant prostate cancer
His: Histidine
PCa: Prostate cancer
IMP: Imidazole propionate
ADT: Androgen deprivation therapy
PBS: Phosphate buffered solution
FBS: Fetal bovine serum
RIPA: Radioimmunoprecipitation
BCA: Bicinchoninic acid
ECL: Enhanced chemiluminescence
HR: Hazard ratio
KEGG: Kyoto encyclopedia of genes and genomes
si-RNA: Small interfering RNA
CCK8: Cell counting Kit-8
