# Supplementary material for: Microbially produced imidazole propionate impairs prostate cancer progression through PDZK1
Source: Mol Med. 2025 Jan 16;31:14. doi: 10.1186/s10020-025-01073-0 (PMC11740605; doi:10.1186/s10020-025-01073-0)
Supplement: Supplementary file 1 — Supplementary Material 1: Figure S1 The effect of IMP on different prostate cells. A Safety assay of IMP on normal prostate cancer cells RWPE-1 cells. B Histogram depicting cell colony counts following stimulation with IMP for PC3 and DU145 cells. C EdU staining plots of DU145 cells after IMP stimulation. Scale bar, 200 μm. D Histogram showing EdU detection results for PC3 and DU145 cells. E Western blot of PDZK1 in tumour and normal tissues. F Apoptosis assay of PC3 and DU145 after 48h of treatment with the corresponding concentrations of IMP. *P < 0.05, **P < 0.01, ***P < 0.001, ****P < 0.0001; ns, not significant. [file 10020_2025_1073_MOESM1_ESM.docx]

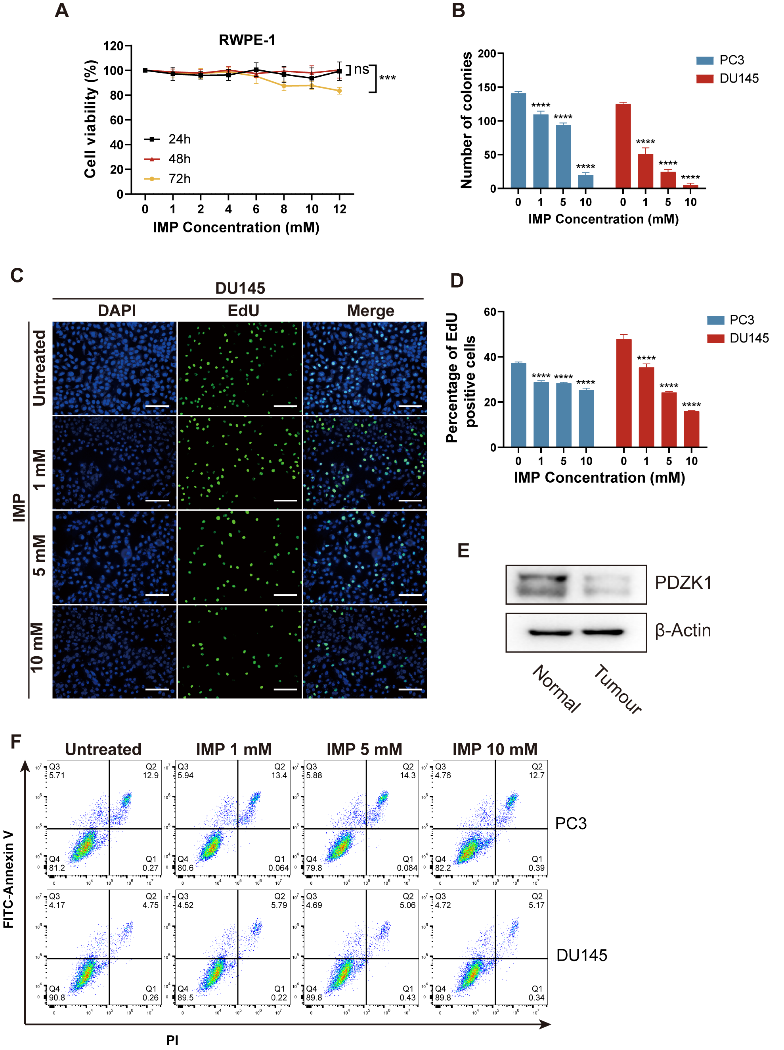


**Figure S1** The effect of IMP on different prostate cells. **A.** Safety assay of IMP on normal prostate cancer cells RWPE-1 cells. **B.** Histogram depicting cell colony counts following stimulation with IMP for PC3 and DU145 cells. **C.** EdU staining plots of DU145 cells after IMP stimulation. Scale bar, 200 μm. **D.** Histogram showing EdU detection results for PC3 and DU145 cells. **E.** Western blot of PDZK1 in tumour and normal tissues. **F.** Apoptosis assay of PC3 and DU145 after 48h of treatment with the corresponding concentrations of IMP. **P* < 0.05, ***P* < 0.01, ****P* < 0.001, *****P* < 0.0001; ns, not significant.
