# Supplementary material for: Microbially produced imidazole propionate impairs prostate cancer progression through PDZK1
Source: Mol Med. 2025 Jan 16;31:14. doi: 10.1186/s10020-025-01073-0 (PMC11740605; doi:10.1186/s10020-025-01073-0)
Supplement: Supplementary file 2 — Supplementary Material 2: Figure S2 Statistical analysis charts. A Histogram of wound healing area for PC3 and DU145. B Histogram of the number of Transwell migrations. C Histogram of cell colony number after knockdown of PDZK1 in PC3 (left) and DU145 (right). D Histogram of Transwell mobility after knockdown of PDZK1 in PC3 (left) and DU145 (right). E Histogram of wound healing rate after knockdown of PDZK1 in PC3 (left) and DU145 (right). F Rate of cell colony formation in rescue experiments with PC3 (left) and DU145 (right). G Histogram of Transwell migration of PC3 (left) and DU145 (right) in rescue experiments. H PDZK1 protein expression levels grayscale analysis in PC3 (left) and DU145 (right) cells treated with different concentrations of IMP. *P < 0.05, **P < 0.01, ***P < 0.001, ****P < 0.0001; ns, not significant. [file 10020_2025_1073_MOESM2_ESM.docx]

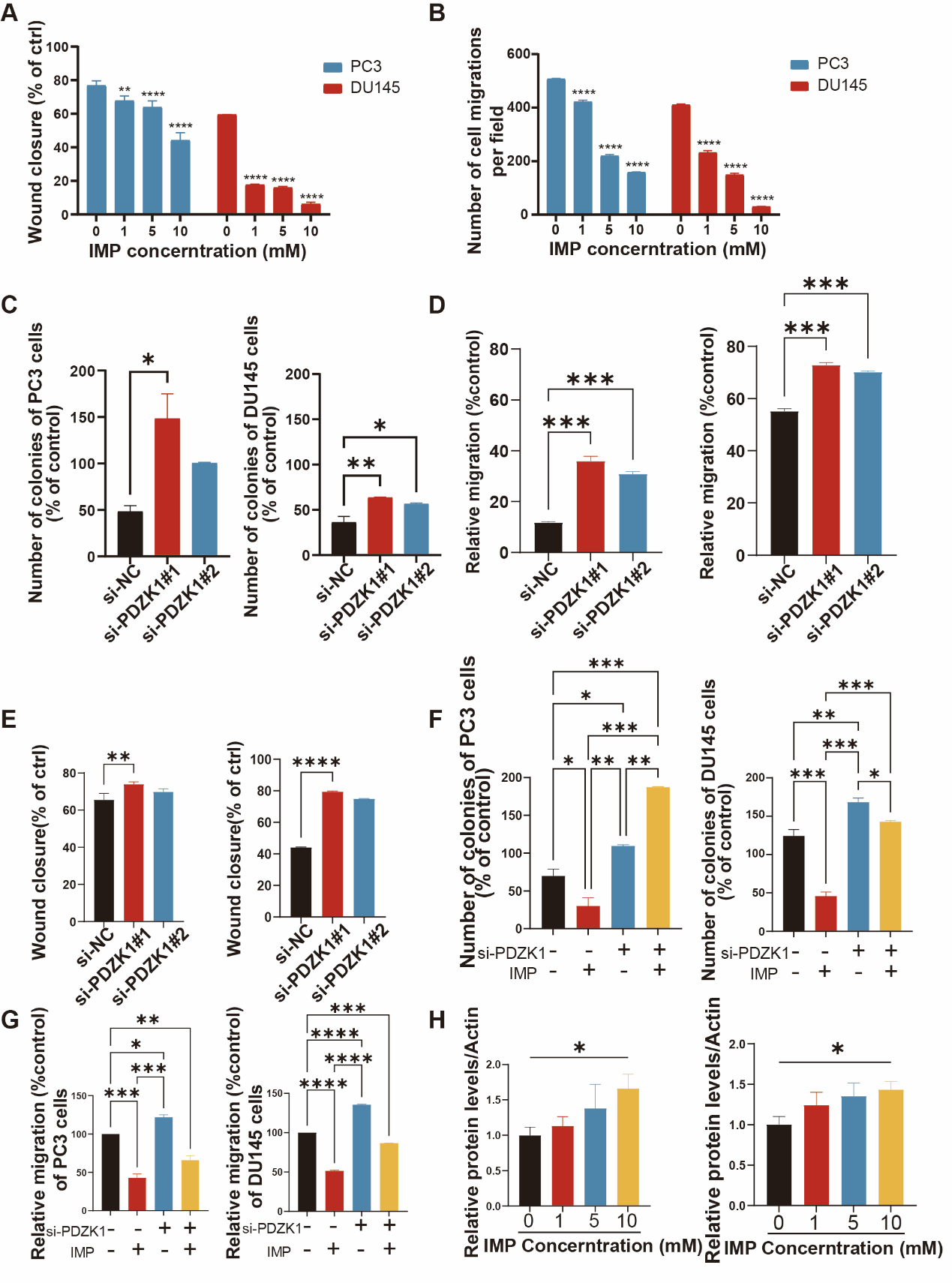


**Figure S2** Statistical analysis charts. **A.** Histogram of wound healing area for PC3 and DU145. **B.** Histogram of the number of Transwell migrations. **C.** Histogram of cell colony number after knockdown of PDZK1 in PC3 (left) and DU145 (right). **D.** Histogram of Transwell mobility after knockdown of PDZK1 in PC3 (left) and DU145 (right). **E.** Histogram of wound healing rate after knockdown of PDZK1 in PC3 (left) and DU145 (right). **F.** Rate of cell colony formation in rescue experiments with PC3 (left) and DU145 (right). **G.** Histogram of Transwell migration of PC3 (left) and DU145 (right) in rescue experiments. **H.** PDZK1 protein expression levels grayscale analysis in PC3 (left) and DU145 (right) cells treated with different concentrations of IMP. **P* < 0.05, ***P* < 0.01, ****P* < 0.001, *****P* < 0.0001; ns, not significant.
