# Supplementary material for: Microbially produced imidazole propionate impairs prostate cancer progression through PDZK1
Source: Mol Med. 2025 Jan 16;31:14. doi: 10.1186/s10020-025-01073-0 (PMC11740605; doi:10.1186/s10020-025-01073-0)
Supplement: Supplementary file 3 — Supplementary Material 3: Figure S3 Analysis of grayscale values and protocols for animal experimentation. A Quantitative analysis of protein phosphorylation of PC3 (left) and DU145 (right) with or without treatment with IMP (10 mM). B Quantitative analysis of protein phosphorylation in PC3 (left) and DU145 (right) cells with or without si-PDZK1 treatment. C Schematic of the mouse experiment: On Day 0, PC3 cells were injected subcutaneously. From Day 3, intraperitoneal injections of either IMP or PBS were administered every two days until the tumors became visible (n = 5 per group). *P < 0.05, **P < 0.01, ***P < 0.001, ****P < 0.0001; ns, not significant. [file 10020_2025_1073_MOESM3_ESM.docx]

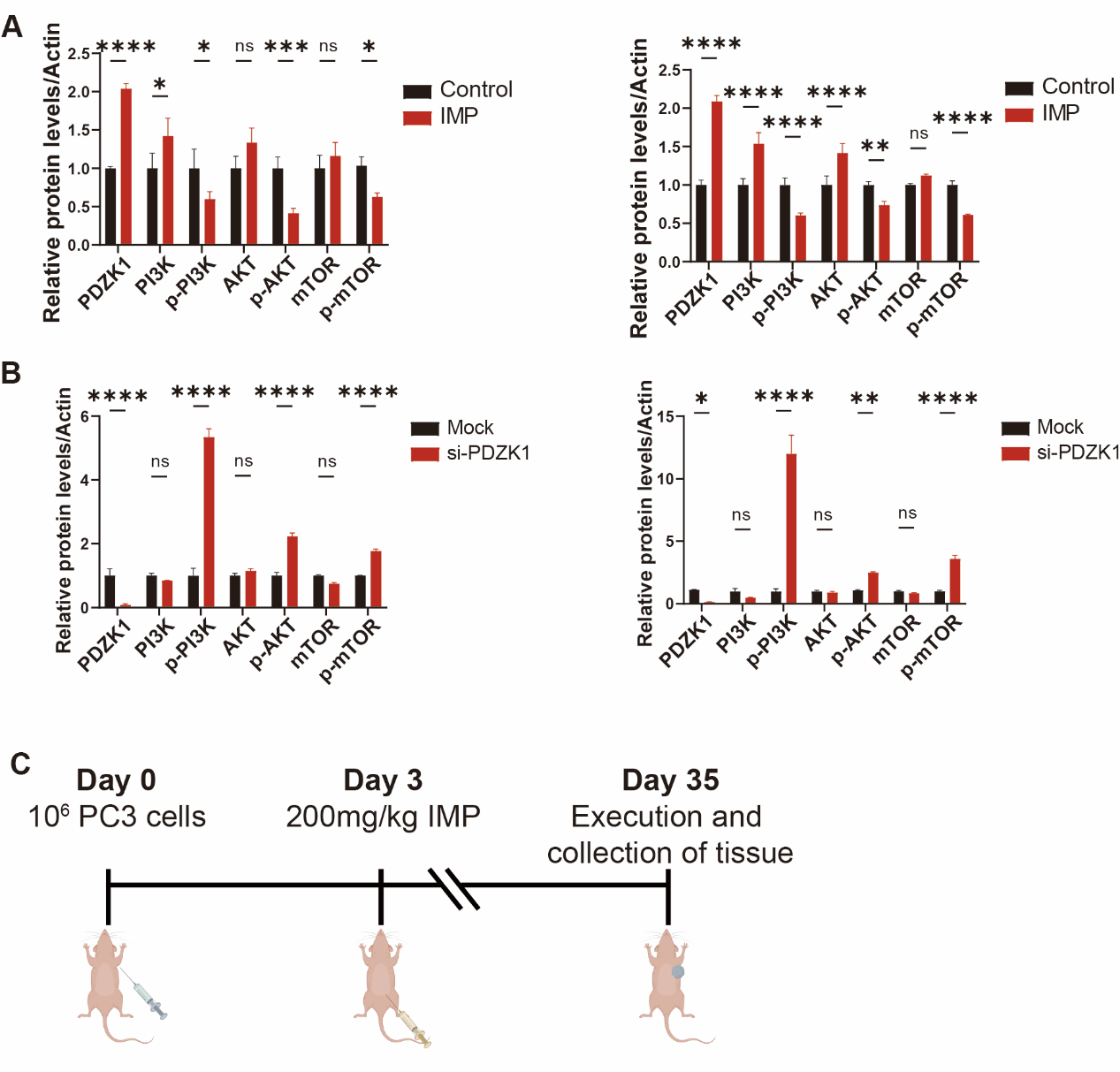


**Figure S3** Analysis of grayscale values and protocols for animal experimentation. **A.** Quantitative analysis of protein phosphorylation of PC3 (left) and DU145 (right) with or without treatment with IMP (10 mM). **B.** Quantitative analysis of protein phosphorylation in PC3 (left) and DU145 (right) cells with or without si-PDZK1 treatment. **C.** Schematic of the mouse experiment: On Day 0, PC3 cells were injected subcutaneously. From Day 3, intraperitoneal injections of either IMP or PBS were administered every two days until the tumors became visible (n = 5 per group). **P* < 0.05, ***P* < 0.01, ****P* < 0.001, *****P* < 0.0001; ns, not significant.
