# Supplementary material for: Microbially produced imidazole propionate impairs prostate cancer progression through PDZK1
Source: Mol Med. 2025 Jan 16;31:14. doi: 10.1186/s10020-025-01073-0 (PMC11740605; doi:10.1186/s10020-025-01073-0)
Supplement: Supplementary file 4 — Supplementary Material 4: Table S1. Primer Sequence List. [file 10020_2025_1073_MOESM4_ESM.docx]

Supplementary Table 1: Primer Sequence List

| Primer name | Primer | Sequence (5'-3') |
| --- | --- | --- |
| PDZK1 | Forward | CCTGAGTGAACGAACAGAGC |
|  | Reverse | TCTCTGCTGGGCTACACTTC |
| NPR3 | Forward | TTCTTCCTACGGAGATGGCT |
|  | Reverse | ACGGTCCTCAGTAGGGTGAC |
| ZBTB16 | Forward | GCACAGTTTTCGAAGGAGGA |
|  | Reverse | GGCCATGTCAGTGCCAGT |
| ALDH1A1 | Forward | GTTAGCTGATGCCGACTTGG |
|  | Reverse | CCCACTCTCAATGAGGTCAAG |
| RARRES2 | Forward | TGGAAGAAACCCGAGTGCAAA |
|  | Reverse | AGAACTTGGGTCTCTATGGGG |
| AKR1B10 | Forward | CCCAGGAGACAGAGGTTATA |
|  | Reverse | GAAATGATTCTGAGTGAGCAGGTAG |
| FER1L4 | Forward | ACACAGTCCTTGTGGGTTCC |
|  | Reverse | CCTGTCTCCTCCATCTCTCC |
| BMF | Forward | GAGGTACAGATTGCCCGAAAG |
|  | Reverse | TTCAAAGCAAGGTTGTGCA |
| Actin | Forward | ACCAACTGGGACGACATGGAGAAA |
|  | Reverse | ATAGCACAGCCTGGATAGCAACG |
| H5174-siPDZK1-1 | Target sequence | GGGUGGACUUGAAAGAGUU(dT)(dT) |
| H5174-siPDZK1-2 | Target sequence | GGGUGAAUGUGCUAGAUGA(dT)(dT) |
| H5174-siPDZK1-3 | Target sequence | GGUUGAGAAGUGUAGCCCA(dT)(dT) |
